# Supplementary material for: Approach to Cohort-Wide Re-Analysis of Exome Data in 1000 Individuals with Neurodevelopmental Disorders
Source: Genes (Basel). 2022 Dec 22;14(1):30. doi: 10.3390/genes14010030 (PMC9858523; doi:10.3390/genes14010030)
Supplement: Supplementary file 1 [file genes-14-00030-s001.zip › File S1&Figure S1-3.pdf]

Supplementary Information

Case reports 1
Methods 6
Figure S3 7
References 8

Case reports

Individual 1, *FOXP4*: In this case, DNA was enriched using SureSelect Human All Exon V6, Agilent. The individual was eight years old at the time of the initial report (in October 2017), clinical features were hearing impairment, ventricular septal defect, flattened epiphysis, disproportionate short stature and craniofacial asymmetry. Family history was unremarkable. The missense variant identified in *FOXP4* gene (NM_001012426.2:c.1540G>A, p.Ala514Thr) was classified as pathogenic based on ACMG criteria (PS2, PS3, PS4_MOD, PM2_SUP, PP3). The *FOXP4* gene follows an autosomal dominant mode of inheritance, and the variant occurred heterozygous *de novo*. This variant has been reported in a publication by Snijders Blok et al. [1]. Here, all affected individuals were characterised by short stature, muscular hypotonia, delayed motor and speech development, ptosis and strabismus. They had normal intelligence. Also, variable congenital malformations were diagnosed in the cohort. This variant was initially not reported because the diagnostic relevance of the gene was not yet known. The first publication on the *FOXP4* gene was published in March 2021[1], which is 53 months after the initial report date.

Individual 2, *KMT2C*: In this case, DNA was enriched using SureSelect Human All Exon V6, Agilent. The initial analysis was performed in September 2017, when the individual's age was ten years. She presented with hypothyroidism, mild intellectual disability, mild abnormality of facial shape and mild short stature. Family history was unremarkable in this regard. No other clinical examination findings were available. The loss of function-variant found in the *KMT2C* gene (NM_170606.3:c.1829_1830delCA, p.Thr610Serfs*4) was classified as pathogenic (PVS1, PS2_MOD, PS4_SUP, PM2_SUP) according to the ACMG criteria. Kleefstra syndrome 2 is associated with *KMT2C* gene, which follows an autosomal dominant mode of inheritance. Koemans et al. reported a cohort consisting of five individuals with *de novo* loss of function variants; all individuals had mild to severe intellectual disability, delayed language and motor development, and autism or profound developmental disorder [2]. Thus, there is a large overlap with Kleefstra syndrome, which is characterised by intellectual developmental delay with delayed language development, hypotonia, and facial abnormalities, such as micro-/brachycephaly, hypertelorism, midface hypoplasia and prognathism [3]. The variant occurred *de novo*. This variant was not initially evaluated because it was not called by freebayes variant caller (v1.1.0-9-g09d4ecf). For segregation analysis of the variant in *KMT2C* parts of exon 14 of the KMT2C gene (NM_170606.3) were amplified by long-range PCR. Amplicons were

prepared for Nanopore sequencing on a R9.4.1 flowcell using the library preparation kit SQK-NBD112.24 (Oxford Nanopore Technologies). Basecalling, demultiplexing and adapter trimming was performed using guppy v6.1.2 (Oxford Nanopore Technologies). Basecalled reads were mapped against the reference genome hg19 using minimap2 v.2.17-r941 [4] and visualised with IGV browser [5], see Figure S1.

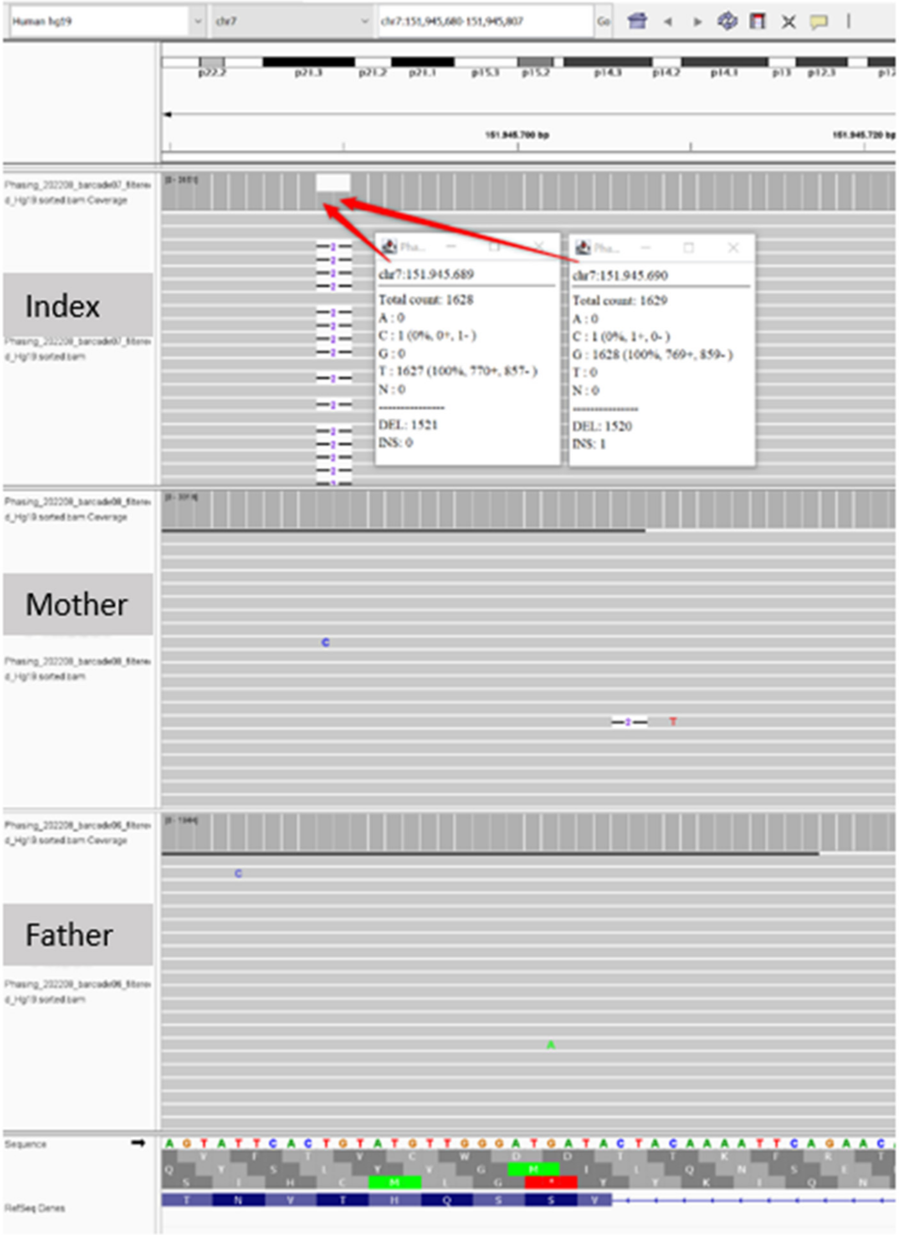

Figure S1: Exemplary screenshot from IGV of Nanopore sequencing data showing the variant NM_170606.3(*KMT2C*):c.1829_1830delCA, p.Thr610Serfs*4 in Individual 2. The variant is absent in the reads of both parents.

Individual 3, *LMNB1*: In this case, DNA was enriched using BGI Exome capture 59M kit. During pregnancy of the 35-year old mother, in the 32nd week of gestation, microcephaly, agenesis of the corpus callosum, cerebellar hypoplasia and growth retardation of the fetus were

sonographically diagnosed. The mother has had two miscarriages before. The missense variant identified in the *LMNB1* gene (NM_005573.4:c.97A>G, p.Lys33Glu) was classified as pathogenic based on ACMG criteria (PS2_VSTR, PS3, PS4_MOD, PM2_SUP, PP3). Primary autosomal dominant microcephaly 26 is associated with the *LMNB1* gene, which follows an autosomal dominant mode of inheritance. The variant occurred heterozygous *de novo*. This disorder is characterised by the occurrence of marked microcephaly, in association with variable intellectual disability and neurological abnormalities [6]. The presence of the variant was confirmed by Sanger sequencing. This variant was initially not reported because the diagnostic relevance of the gene was not yet known. The first publication on the *LMNB1* gene was published in September 2020 [6], which is nine months after the report date. The variant present in this individual has already been described as *de novo* in two publications [6,7].

Individual 4, *MORC2*: In this case, DNA was enriched using BGI Exome capture 59M kit. The initial evaluation was performed in May 2019, when the individual was two years old. She presented with global developmental delay and microcephaly; family history was unremarkable in this regard. No other clinical findings were available. The missense variant found in the *MORC2* gene (NM_001303256.3:c.79G>A, p.Glu27Lys) was classified as pathogenic according to ACMG criteria (PS2_VSTR, PS3, PS4_MOD, PM2_SUP, PP2). The associated phenotype with *MORC2* gene is a syndrome with developmental delay, growth retardation, dysmorphic facial features, and axonal neuropathy and follows an autosomal dominant mode of inheritance. The variant was present heterozygously, occured *de novo* and was confirmed by Sanger Sequencing. The variant was initially not reported because the diagnostic relevance of the gene was not yet known. The first publication on the *MORC2* gene was published in August 2020 [8], which is 15 months after the initial report. In this publication, five individuals were identified with this variant.

Individual 5, *MSL3*: In this case, DNA was enriched using SureSelect Human All Exon V6, Agilent. The initial evaluation was performed in January 2018, when the individual was three years old. He presented with refractory global developmental delay, seizures, chylothorax and and mid-aortic syndrome; family history was unremarkable in this regard. No other clinical findings were available. The hemizygous, LoF-variant found in the *MSL3* gene (NM_078629.4:c.973_974delAG, p.Gln326Alafs*5) was classified as pathogenic according to the ACMG criteria (PVS1, PS2, PS4_SUP, PM2_SUP). Basilicata-Akhtar syndrome is associated with *MSL3*, which follows an X-linked dominant inheritance. Basilicata-Akhar syndrome is characterised by developmental delays and mild to severe intellectual disability. In addition, autism, changes in muscle tone (hypotonia or spasticity), macrocephaly, hearing impairment, and gastrointestinal problems have been reported [9,10]. The variant occurred *de novo*. No validation analysis was performed as the variant had good coverage. At the time of the initial analysis the diagnostic relevance of the gene was not yet known. The first publication on the *MSL3* gene appeared in October 2018 [10], which is nine months after the initial report. The variant present in this individual was already described as *de novo* in another publication [9].

Individual 6, *MTOR*: In this case, DNA was enriched using BGI Exome capture 59M kit. The initial evaluation was performed in September 2017, when the individual was three years old. He presented with global developmental delay and macrocephaly; family history was unremarkable in this regard. No other clinical findings were available. The heterozygous, missense-variant found in the *MTOR* gene (NM_004958.4:c.5911G>A, p.Ala1971Thr) was classified as likely pathogenic according to the ACMG criteria (PS2, PS4_SUP, PM2_SUP, PM5_SUP, PP2, PP3). The associated OMIM Phenotype (#616638) is Smith-Kingsmore Syndrome, an autosomal dominant disorder that is characterised by developmental delay, seizures and macrocephaly [11,12]. The variant occurred *de novo*. This variant was not initially evaluated because it was not called by freebayes variant caller (v1.1.0-9-g09d4ecf), probably because it is a mosaicism of 11 %. Gonadal mosaicisms in MTOR are described in the literature as causative for Smith-Kingsmore Syndrome [12]. Validation by Sanger sequencing is pending and currently being performed.

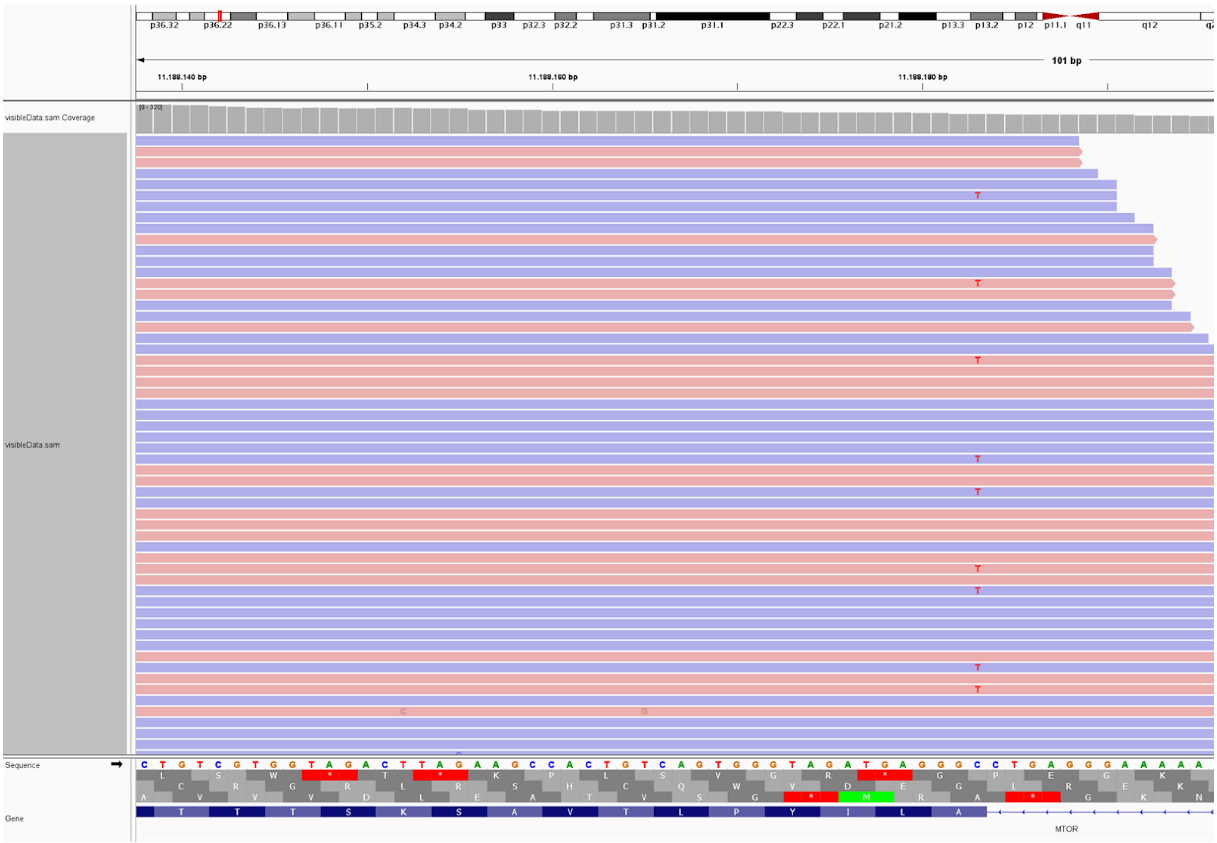

Figure S2: Exemplary screenshot from IGV showing the variant NM_004958.4:c.5911G>A, p.Ala1971Thr in Individual 6. Twenty-one of 196 reads show a thymine at position Chr1:11188183 in hg19 (i.e. Chr1:11128126 in hg38) instead of the expected cytosine. This translates to a mosaicism of 11%. Exon structure of MTOR is visualised in the lowest track. The variant changes the first base of exon 43/58 in NM_004958.4.

Individual 7, *SORD*: In this case, DNA was enriched using BGI Exome capture 59M kit. The initial evaluation was performed in February 2020, and the individual was 25 years old at that time. He presented with leg pain, existing since the age of 17 and occurring mainly during

walking, gait disturbance and atrophy of the legs. The family history was unremarkable, the parents are consanguineous. The identified LoF variant in the *SORD* gene (NM_003104.5:c.757del, p.Ala253Glnfs*27) was classified as pathogenic based on the ACMG criteria (PVS1, PS3, PM3_VSTR). The associated phenotype with *SORD* gene is sorbitol dehydrogenase deficiency with peripheral neuropathy, which follows an autosomal recessive mode of inheritance. The variant occurred homozygous and was validated by Sanger sequencing. This disorder is characterised by gait disturbances, and weakness of the distal lower extremities [13]. The variant was initially not identified because the diagnostic relevance of the gene was not yet known. The first publication on the *SORD* gene was published in May 2020 [13]. Biallelic variants in the *SORD* gene are causative for the most common recessive form of hereditary neuropathies, and this variant is the most common to be detected in affected individuals [13].

Individual 8, *SDHC*: In this case, DNA was enriched using SureSelect Human All Exon V6, Agilent. The initial evaluation was performed in December 2017, when the individual was three years old. She presented with mild developmental delay, seizures, heterotopia, oral cleft, tall stature and obesity; family history was unremarkable in this regard. The missense variant found in the *SDHC* gene (NM_003001.5:c.377A>G, p.Tyr126Cys) was classified as likely pathogenic according to ACMG criteria (PS4_MOD, PM1, PM2_SUP, PP3). The associated phenotype with *SDHC* gene is paraganglioma-pheochromocytoma syndrome, which follows an autosomal dominant mode of inheritance. The variant was present heterozygously and inherited paternally. Hereditary paraganglioma-pheochromocytoma syndrome is characterized by the development of paragangliomas and pheochromocytomas, which may be associated with catecholamine excess or displacement/obstruction of other structures, depending on their origin. Associated symptoms are persistent or paroxysmal elevations of blood pressure, headache, episodic sweating, severe palpitations, pallor, and trepidation or anxiety [14]. The *SDHC* gene is found in a list of 73 genes listed by the ACMG as additional findings [15]. Consent to report these additional findings is required and available here. The variant was initially not reported because it was not called by variant caller freebayes (v1.1.0-9-g09d4ecf).

Individual 9, *TTN*: In this case, DNA was enriched using TWIST Human Core Exome Kit. The initial evaluation was performed in September 2020, and the individual was 18 years old. She presented with panhypopituitarism, developmental delay, patent ductus arteriosus, scoliosis, short stature and median cleft lip and palate. The family history was unremarkable. In trio-exome diagnostics, a *DIPS1*-related disorder could be identified based on compound-heterozygous variants (p.Met453Leu, p.Arg1078His). In reanalysis, additionally a LoF-variant in the *TTN* gene (NM_001267550.2:c.80762_80765delAACA, p.Lys26921Argfs*5) was identified and classified as likely pathogenic based on ACMG criteria (PVS1, PM2_SUP). The associated phenotype is dilated cardiomyopathy, which follows an autosomal dominant mode of inheritance. The variant was present heterozygously and maternally inherited. Truncating variants in *TTN* are on ACMG secondary findings list [15,16] since August 2021. Studies showed that variants associated with dilatative cardiomyopathy are overrepresented in A-band [17], and this variant lies within. Consent for secondary findings is available.

Methods

Reprocessing of BAM files

All raw sequencing data in bcl format was initially processed using the cloud based "varfeed" pipeline (https://www.limbus-medtec.com/; Limbus Medical Technologies GmbH, Rostock, Germany). For re-processing, we downloaded the BAM (Binary SAM (Sequence Alignment Map)) files previously aligned to hg19 from the Amazon S3 bucket using the AWS CLI tools (https://aws.amazon.com/de/cli/) and custom shell scripts parallelized using GNU Parallel [18].

These BAM files were then used as input for a new alignment pipeline. We used the Broad hg38 version from their "broad-resource-bundle" (https://console.cloud.google.com/storage/browser/genomics-public-data/resources/broad/hg38/v0) as a new reference downloaded using the gsutil application (https://cloud.google.com/storage/docs/gsutil). We designed a Nextflow [19] workflow based on GATK (Genome Analysis Toolkit) [20] recommendations for new alignment of BAM files. First, we created an unmapped BAM file using the Picard Tools (version 2.25. 0; https://broadinstitute.github.io/picard/) "RevertSam" command. Next, we converted the reverted BAM to interleaved FASTQ format files using the "SamToFastq" Picard tool, aligned the reverted BAM using the BWA-MEM [21] Sentieon (https://www.sentieon.com/; release 202010) [22] implementation version 0.7.17-r1188, and finally, we merged the BAM alignment using the Picard "MergeBamAlignment" tool. The duplicates in the merged BAM files were then identified using the Sentieon deduplication tools "LocusCollector" and "Dedup". In the subsequent step, we calibrated the base quality using the "QualCal" tool from Sentieon. Lastly, we used the Crumble algorithm [23] to reduce the size of the BAM files and make the final hg38 BAM files that could be used for subsequent analysis steps.

Variant calling from reprocessed hg38 BAM files

To generate per-sample genomic VCFs (GVCFs), the final BAM files were called individually using the Sentieon implementation of the GATK HaplotypeCaller algorithm called "Haplotyper" [24]. The Sentieon "GVCFtyper" command was used to create a single sample VCF from the GVCF of each sample. Prior to variant score calibration, this single sample VCF was subset to the respective exome design's target BED (Browser Extensible Data) file and split into two files containing SNPs and other variants (indels, MNVs) using the GATK (version 4.2.0.0) "SelectVariants" command. These were used to recalculate the variant quality scores with the recommended settings of GATK "VariantRecalibrator" in the Sentieon implementation "VarCal" and to apply the models using Sentieon "ApplyVarCal." For each sample, the final calibrated and ontarget VCF files were generated by merging the two VCFs using Picard's "MergeVcfs" command, which employed gzip compression for the output and Tabix indexes

Variant annotation

SNVs were merged on cohort level and annotated using vsWarehouse (Golden Helix, Bozeman, Montana, USA). Annotation sources included: RefSeq, OMIM, gnomAD, ClinVar, dbscSNV splice predictor, dbNSFP functional predictor, GERP++, ACMG Auto Classification

(GoldenHelix), as well as counts of all observed alleles of the respective cohort (gnomAD release 2.0.1). Cohort specific SNV datasets have been exported, rearranged and filtered using custom scripts to obtain final SNV sets for manual evaluation.

Sanger sequencing for validation of novel variants

PCR was done using DreamTaq Hot Start DNA Polymerase (ThermoScientific) or HotStartTaq Plus (Qiagen). Sanger sequencing was performed using PCR product after clean-up using Exo SAP-IT Express (Applied Biosystems) and internal oligos. Sequencing reaction was done using Big Dye Terminator v3.1 Cycle Sequencing kit and Big Dye Terminator v1.1, v3.1 5x Sequencing Buffer. Final clean-up was done using ethanol. Electrophoresis was performed on an ABI3500 capillary elektrophoresis instrument (Thermo Scientific). Data was assessed using Sequence Pilot Software (JSI medical systems).

Nanopore sequencing for validation of novel variants

Method is described in the case report of Individual 2.

HPOsim score

Basically, the HPOsim score reflects the similarity of two HPO-term sets considering also the relevance of HPO-terms. Essential for its calculation is the information content [25] of HPO-terms that exploits the frequency of each HPO-term in the gene data of the HPO database. The more genes an HPO-term is associated with, the lower is its information content. Considering the information content of two HPO-terms and the information content of their most informative common ancestor term, the similarity of two HPO-terms is calculated based on Lin's method [26]. Finally, by calculating all pairwise term similarities, the similarity of two HPO-term sets is calculated based on the Best-Match-Average strategy [27] resulting in a score in [0,1], whereby a higher value is better.

Figure S3

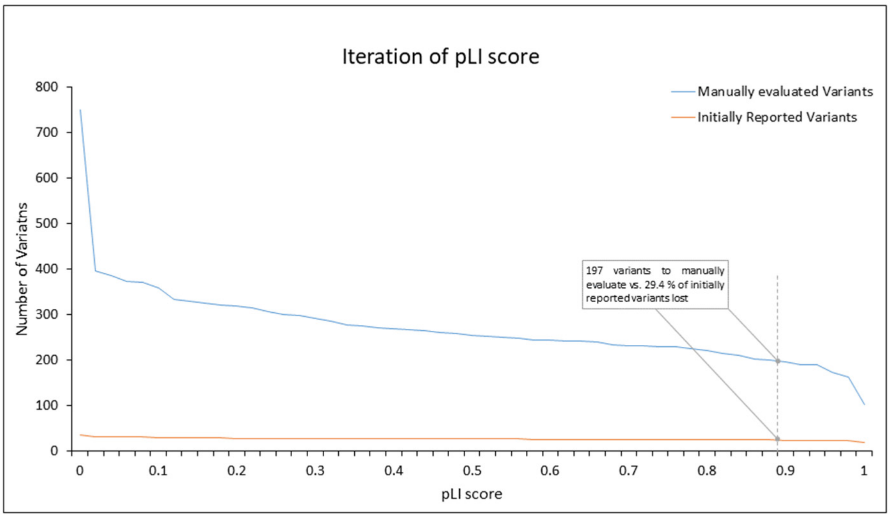

Number of variants to be manually evaluated (blue) and of initially reported (L)P variants (orange) based on pLI score thresholds as single filter criterion. Depicted line marks the cut-off of 0.9

References

1. Snijders Blok, L.; Vino, A.; den Hoed, J.; Underhill, H.R.; Monteil, D.; Li, H.; Reynoso Santos, F.J.; Chung, W.K.; Amaral, M.D.; Schnur, R.E.; et al. Heterozygous Variants That Disturb the Transcriptional Repressor Activity of FOXP4 Cause a Developmental Disorder with Speech/Language Delays and Multiple Congenital Abnormalities. *Genet Med* **2021**, *23*, 534–542, doi:10.1038/s41436-020-01016-6.

2. Koemans, T.S.; Kleefstra, T.; Chubak, M.C.; Stone, M.H.; Reijnders, M.R.F.; Munnik, S. de; Willemsen, M.H.; Fenckova, M.; Stumpel, C.T.R.M.; Bok, L.A.; et al. Functional Convergence of Histone Methyltransferases EHMT1 and KMT2C Involved in Intellectual Disability and Autism Spectrum Disorder. *PLOS Genetics* **2017**, *13*, e1006864, doi:10.1371/journal.pgen.1006864.

3. Kleefstra, T.; de Leeuw, N. Kleefstra Syndrome. In *GeneReviews*®; Adam, M.P., Everman, D.B., Mirzaa, G.M., Pagon, R.A., Wallace, S.E., Bean, L.J., Gripp, K.W., Amemiya, A., Eds.; University of Washington, Seattle: Seattle (WA), 1993.

4. Li, H. Minimap2: Pairwise Alignment for Nucleotide Sequences. *Bioinformatics* **2018**, *34*, 3094–3100, doi:10.1093/bioinformatics/bty191.

5. Robinson, J.T.; Thorvaldsdóttir, H.; Winckler, W.; Guttman, M.; Lander, E.S.; Getz, G.; Mesirov, J.P. Integrative Genomics Viewer. *Nat Biotechnol* **2011**, *29*, 24–26, doi:10.1038/nbt.1754.

6. Cristofoli, F.; Moss, T.; Moore, H.W.; Devriendt, K.; Flanagan-Steet, H.; May, M.; Jones, J.; Roelens, F.; Fons, C.; Fernandez, A.; et al. De Novo Variants in LMNB1 Cause Pronounced Syndromic Microcephaly and Disruption of Nuclear Envelope Integrity. *The American Journal of Human Genetics* **2020**, *107*, 753–762, doi:10.1016/j.ajhg.2020.08.015.

7. Parry, D.A.; Martin, C.-A.; Greene, P.; Marsh, J.A.; Ambrose, J.C.; Arumugam, P.; Baple, E.L.; Bleda, M.; Boardman-Pretty, F.; Boissiere, J.M.; et al. Heterozygous Lamin B1 and Lamin B2 Variants Cause Primary Microcephaly and Define a Novel Laminopathy. *Genetics in Medicine* **2021**, *23*, 408–414, doi:10.1038/s41436-020-00980-3.

8. Guillen Sacoto, M.J.; Tchasovnikarova, I.A.; Torti, E.; Forster, C.; Andrew, E.H.; Anselm, I.; Baranano, K.W.; Briere, L.C.; Cohen, J.S.; Craigen, W.J.; et al. De Novo Variants in the ATPase Module of MORC2 Cause a Neurodevelopmental Disorder with Growth Retardation and Variable Craniofacial Dysmorphism. *Am J Hum Genet* **2020**, *107*, 352–363, doi:10.1016/j.ajhg.2020.06.013.

9. Brunet, T.; McWalter, K.; Mayerhanser, K.; Anbouba, G.M.; Armstrong-Javors, A.; Bader, I.; Baugh, E.; Begtrup, A.; Bupp, C.P.; Callewaert, B.L.; et al. Defining the Genotypic and Phenotypic Spectrum of X-Linked MSL3-Related Disorder. *Genet Med* **2021**, *23*, 384–395, doi:10.1038/s41436-020-00993-y.

10. Basilicata, M.F.; Bruel, A.-L.; Semplicio, G.; Valsecchi, C.I.K.; Aktaş, T.; Duffourd, Y.; Rumpf, T.; Morton, J.; Bache, I.; Szymanski, W.G.; et al. De Novo Mutations in MSL3 Cause an X-Linked Syndrome Marked by Impaired Histone H4 Lysine 16 Acetylation. *Nat Genet* **2018**, *50*, 1442–1451, doi:10.1038/s41588-018-0220-y.

11. Smith, L.; Saunders, C.; Dinwiddie, D.; Atherton, A.; Miller, N.; Soden, S.; Farrow, E.; Abdelmoity, A.; Kingsmore, S. Exome Sequencing Reveals De Novo Germline Mutation of the Mammalian Target of Rapamycin (MTOR) in a Patient with Megalencephaly and Intractable Seizures. *Journal of Genomes and Exomes* **2013**, *2013*, 63–72, doi:10.4137/JGE.S12583.

12. Mroske, C.; Rasmussen, K.; Shinde, D.N.; Huether, R.; Powis, Z.; Lu, H.-M.; Baxter, R.M.; McPherson, E.; Tang, S. Germline Activating MTOR Mutation Arising through Gonadal Mosaicism in Two Brothers with Megalencephaly and Neurodevelopmental Abnormalities. *BMC Med Genet* **2015**, *16*, 102, doi:10.1186/s12881-015-0240-8.

13. Cortese, A.; Zhu, Y.; Rebelo, A.P.; Negri, S.; Courel, S.; Abreu, L.; Bacon, C.J.; Bai, Y.; Bis-Brewer, D.M.; Bugiardini, E.; et al. Biallelic Mutations in SORD Cause a Common and Potentially Treatable Hereditary Neuropathy with Implications for Diabetes. *Nat Genet* **2020**, *52*, 473–481, doi:10.1038/s41588-020-0615-4.

14. Else, T.; Greenberg, S.; Fishbein, L. Hereditary Paraganglioma-Pheochromocytoma Syndromes. In *GeneReviews®*; Adam, M.P., Everman, D.B., Mirzaa, G.M., Pagon, R.A., Wallace, S.E., Bean, L.J., Gripp, K.W., Amemiya, A., Eds.; University of Washington, Seattle: Seattle (WA), 1993.

15. Miller, D.T.; Lee, K.; Chung, W.K.; Gordon, A.S.; Herman, G.E.; Klein, T.E.; Stewart, D.R.; Amendola, L.M.; Adelman, K.; Bale, S.J.; et al. ACMG SF v3.0 List for Reporting of Secondary Findings in Clinical Exome and Genome Sequencing: A Policy Statement of the American College of Medical Genetics and Genomics (ACMG). *Genetics in Medicine* **2021**, *23*, 1381–1390, doi:10.1038/s41436-021-01172-3.

16. Roberts, A.M.; Ware, J.S.; Herman, D.S.; Schafer, S.; Baksi, J.; Bick, A.G.; Buchan, R.J.; Walsh, R.; John, S.; Wilkinson, S.; et al. Integrated Allelic, Transcriptional, and Phenomic Dissection of the Cardiac Effects of Titin Truncations in Health and Disease. *Sci Transl Med* **2015**, *7*, 270ra6, doi:10.1126/scitranslmed.3010134.

17. Herman, D.S.; Lam, L.; Taylor, M.R.G.; Wang, L.; Teekakirikul, P.; Christodoulou, D.; Conner, L.; DePalma, S.R.; McDonough, B.; Sparks, E.; et al. Truncations of Titin Causing Dilated Cardiomyopathy. *N Engl J Med* **2012**, *366*, 619–628, doi:10.1056/NEJMoa1110186.

18. Tange, O. *GNU Parallel 2018*; Ole Tange, 2018; ISBN 978-1-387-50988-1.

19. Di Tommaso, P.; Chatzou, M.; Floden, E.W.; Barja, P.P.; Palumbo, E.; Notredame, C. Nextflow Enables Reproducible Computational Workflows. *Nat Biotechnol* **2017**, *35*, 316–319, doi:10.1038/nbt.3820.

20. McKenna, A.; Hanna, M.; Banks, E.; Sivachenko, A.; Cibulskis, K.; Kernytsky, A.; Garimella, K.; Altshuler, D.; Gabriel, S.; Daly, M.; et al. The Genome Analysis Toolkit: A MapReduce Framework for Analyzing next-Generation DNA Sequencing Data. *Genome Res* **2010**, *20*, 1297–1303, doi:10.1101/gr.107524.110.

21. Li, H. Aligning Sequence Reads, Clone Sequences and Assembly Contigs with BWA-MEM 2013.

22. Freed, D.; Aldana, R.; Weber, J.A.; Edwards, J.S. *The Sentieon Genomics Tools - A Fast and Accurate Solution to Variant Calling from next-Generation Sequence Data*; Bioinformatics, 2017;

23. Bonfield, J.K.; McCarthy, S.A.; Durbin, R. Crumble: Reference Free Lossy Compression of Sequence Quality Values. *Bioinformatics* **2019**, *35*, 337–339, doi:10.1093/bioinformatics/bty608.

24. Kendig, K.I.; Baheti, S.; Bockol, M.A.; Drucker, T.M.; Hart, S.N.; Heldenbrand, J.R.; Hernaez, M.; Hudson, M.E.; Kalmbach, M.T.; Klee, E.W.; et al. Sentieon DNASeq Variant Calling Workflow Demonstrates Strong Computational Performance and Accuracy. *Frontiers in Genetics* **2019**, *10*.

25. Resnik, P. Using Information Content to Evaluate Semantic Similarity in a Taxonomy. In *Proceedings of the 14th international joint conference on Artificial intelligence - Volume 1*; IJCAI'95; Morgan Kaufmann Publishers Inc.: San Francisco, CA, USA, 1995; pp. 448–453 ISBN 978-1-55860-363-9.

26. Lin, D. An Information-Theoretic Definition of Similarity. In *Proceedings of the Fifteenth International Conference on Machine Learning*; ICML '98; Morgan Kaufmann Publishers Inc.: San Francisco, CA, USA, 1998; pp. 296–304 ISBN 978-1-55860-556-5.

27. Deng, Y.; Gao, L.; Wang, B.; Guo, X. HPOSim: An R Package for Phenotypic Similarity Measure and Enrichment Analysis Based on the Human Phenotype Ontology. *PLOS ONE* **2015**, *10*, e0115692, doi:10.1371/journal.pone.0115692.
